# Model predictive control based MLP-ANN to enhance tracking response with energy saving of EV drive cycles using five-phase IPMSM

**DOI:** 10.1371/journal.pone.0340199

**Published:** 2026-02-05

**Authors:** Ahmed M. Hassan, Hamada Esmaiel, Mohammed M. Alammar, Mohamed Eladly Metwally

**Affiliations:** 1 Department of Electrical Power and Machines Engineering, Faculty of Engineering, Benha University, Cairo, Egypt; 2 Department of Electrical Power and Machines Engineering, Higher Institute of Engineering (HIE), El-Shorouk Academy, El-Shorouk City, Egypt; 3 Electrical Engineering Department, College of Engineering, King Khalid University, Abha, Saudi Arabia; Tanta University Faculty of Engineering, EGYPT

## Abstract

Speed tracking control (STC) and energy saving of an electric vehicle (EV) play a crucial role in the stability and effectiveness of the operating performance of an EV drive system (EVDS). This paper proposes a novel STC and energy saving methodology for an EVDS that is tested by the European drive cycle (ECE-15) and customized inspection and maintenance drive cycle (custom IM240). The EVDS adopted the 5-ph interior permanent magnet synchronous motor (IPMSM) due to its benefits like high efficiency, compact size, low noise, reliability, reduced torque ripples, increased power density, and improved fault tolerance. A multilayer perceptron (MLP) - artificial neural network (ANN) is utilized to tune the PI controller online in the drive system (DS). The gating pulses of the 5-phase voltage source inverter (VSI) are accurately generated using an MPC based on a desired cost function to reduce current harmonics and thus torque ripples. A comparative study between the online tuning using the MLP-ANN and offline tuning using the transit search optimization (TSO) technique is presented. The process of comparison includes the percentage overshoot, mean square error (MSE), integral absolute error (IAE), and percentage energy saving. The result of the comparison proves the effectiveness of the proposed control methodology that gives superior speed tracking performance of the EVDS and attains energy saving. The attained energy saving across a long period results in cost reduction of charging the batteries and increasing their lifetime.

## 1 Introduction

Recently, air pollution reduction has received a lot of attention from the global community. Electric vehicles play an important role in the reduction of air pollution due to the absence of carbon emissions compared with traditional fuel vehicles [1,2]. To enhance the performance of the EV, a highly efficient drive system, effective speed tracking control, and energy-saving methodology should be introduced. The core of the EV drive system (EVDS) is the traction motor. There are several motors used in the EVDSs. The most used type is the IPMSM due to its benefits [2,3]. These benefits encompass excellent performance, low losses, compact size, etc. [2,4,5]. Because of their inherent benefits, multiphase motor drive systems (DSs) are becoming a promising alternative to 3-phase motor DSs. These benefits include reduced torque ripples and improved fault tolerance [6,7]. MPC is gaining prominence as a control method for DSs, showcasing exceptional performance and optimization [8].

The STC of an EV plays a vital role in the stability and effectiveness of the operating performance of the EV. Consequently, improving the speed response of an EV is considered to be an important subject. To the knowledge of the authors of this paper, there are no published papers that have studied the STC for an EV drive system utilizing a 5-phase IMPSM, MPC, and ANN online tuning to be driven according to different drive cycles. The analysis focuses on improving the speed tracking response that gives lower values of MSE, IAE, and speed overshoots across the whole operating period.

Literature survey of control methods regarding the adopted drive system is explored as follows:

Reference [9] introduced a sensorless control technique utilizing the 3rd harmonic space for a 5-ph PMSM. A direct torque MPC method for a five-phase PMSM was presented in [10]. In reference [11], a model predictive torque control (MPTC) technique was introduced for a 5-phase PMSM. Furthermore, reference [12] introduced an MPTC method that incorporates additional weighting factors to effectively reduce current harmonics and torque ripple. Reference [13] introduced a model-free predictive current control approach that leverages an ultra-local model and motor outputs for 5-phase PMSM DSs. Additionally, reference [14] proposed an MPCC approach for a five-phase PMPSM that employs voltage vector pre-selection.

Reference [15] evaluated different optimization methods to identify the best PI controller gains for a 3-phase PMSM DS. Reference [16] introduced an NN-based MPC method for regulating the speed of a 3-phase PMSM. Likewise, reference [17] introduced a control method using a BP ANN for a 3-phase PMSM. Reference [18] presented sensorless control of a PMSM by employing an adaptive speed observer (ASO). References [19–21] introduced a control method for a 3-phase PMSM, using an MPC-based ANN. Reference [22] introduced an MPC methodology for a 3-phase PMSM that employs dual-vector-based PSO.

Reference [23] introduced a deep reinforcement learning (DRL) methodology for controlling the speed of a 3-phase PMSM DS. This DRL control notably enhanced system performance, particularly in scenarios involving load variations. Reference [24] introduced DRL-based MPC to boost the efficiency of EVs. Reference [25] proposed a control methodology for a 3-phase PMSM using several optimization techniques and RL. Reference [26] suggested an RL control for a 3-phase PMSM, based on the twin delay deep deterministic (TD3) approach. Reference [27] compared the traditional PID method and an RL to control a PMSM, employing vector control (VC).

Reference [28] presented a combination of a hybrid adaptive nonlinear control (HANC) and a deadbeat observer (DO) to enhance the efficiency of a 3-phase PMSM, accommodating changes in load conditions and motor parameters. An adaptive fuzzy proportional-integral (AFPI) control scheme for a 3-phase PMSM was introduced in [29]. Reference [30] proposed an adaptive extended state observer to address peak issues and compensate for mismatched disturbances in a non-cascade-controlled PMSM. The impact of different hybrid EV powertrain options, including fuel cells, batteries, and supercapacitors, on component efficiency was investigated in [31]. Reference [32] introduced an adaptive speed control method utilizing a recurrent Elman neural network (RENN) to ensure good performance ST regardless of system uncertainties in the sensorless PMSM servo drive. A fractional order PID (FOPID) controller based on an adaptive neuro-fuzzy inference system for EV ST, utilizing a DC motor, was introduced in [33]. The performance, energy consumption, and robustness of the controller were evaluated using the New European Driving Cycle (NEDC) test. Reference [34] developed a simulation model for EV traction applications using a model-based design approach. The study incorporated a 3-phase IPMSM. A control algorithm for 3-phase PMSM-based EVs, incorporating the advantages of conventional proportional resonance (PR) and PI controllers, was introduced in Reference [35]. Reference [36] provided modeling and a comparative dynamic analysis of an FOC three-phase PMSM torque drive, utilizing both a hysteresis current controller and a PWM-operated current controller for energy-efficient EVs. Various EV batteries performance was evaluated in Reference [37] using different drive cycles. Reference [38] introduced a control methodology for the DS of an EV’s PMSM. It utilized a robust non-linear MPC in a cascaded structure, combined with SVPWM. A drive control methodology for a three-phase PMSM powered by a multi-level inverter for EV applications was presented in [39]. The study also involved designing an active damping and current controller to deliver a rapid and precise torque response to the induced torsional moments. A machine learning (ML) based controller for a 3-phase PMSM DS was presented in [40]. Reference [41] presented an RL control methodology based on the TD3 technique for tuning cascaded PI controllers in a DS utilizing 5-phase PMSM and MPC.

In this research, a new speed tracking control and energy saving methodology for the EV drive system is proposed and tested by ECE-15 and IM240 drive cycles. The adopted drive system is composed of an EV, 5-phase VSI, 5-phase IPMSM, PI controller, and MPC. The PI controller is tuned using an MLP-ANN online tuning to generate the reference torque signal. This overcomes any load variation compared with offline tuning. The load variation is represented by the test drive cycles. The reference torque signal is utilized to obtain the reference direct and quadrature current to verify the operation of the 5-ph IMPSM at MTPA to attain high efficiency and thus low losses. The MPC is used in the drive system to generate the gating pulses for the 5-phase VSI according to the desired reference direct and quadrature currents. To validate the presented control method, simulation results are generated using MATLAB Simulink. To indicate the effectiveness of the proposed control methodology, several results are obtained when an offline tuning for the PI controller is compared with those obtained when the ANN online tuning is used. The process of comparison includes the percentage overshoot, MSE, IAE, and energy saving performance. In the offline tuning, the PI controller gains are obtained using a metaheuristic optimization technique called Transit Search.

The main contributions of this paper can be summarized as follows:

- A methodology of control for a high-performance EV drive system is proposed and verified using two drive cycles to enhance the speed tracking response and to attain energy saving.
- A comparative analysis is carried out between the presented methodology of control, which is based on MLP-ANN online tuning, and the conventional offline tuning method to prove the effectiveness of the proposed methodology compared with the conventional method.
- The presented control methodology can be considered as a saving energy method.
- A MATLAB SIMULINK is carried out for the EVDS under consideration to validate the presented control method.

The structure of the remaining sections of this paper is as follows: Section 2 introduces the EVDS model. Section 3 introduces an explanation of the MPC. Section 4 covers the MLP-ANN online tuning. Section 5 explains the transit search optimization technique. Section 6 explores the results. Section 7 presents the summary of the findings.

## 2 Modeling of the EV drive system

**Fig 1:**
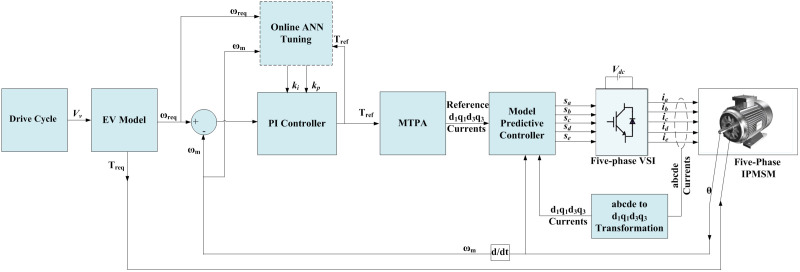
EV drive system under consideration.

In the following subsections, each part of the EVDS shown in Fig 1 will be introduced.

### 2.1 Drive cycle model

**Fig 2:**
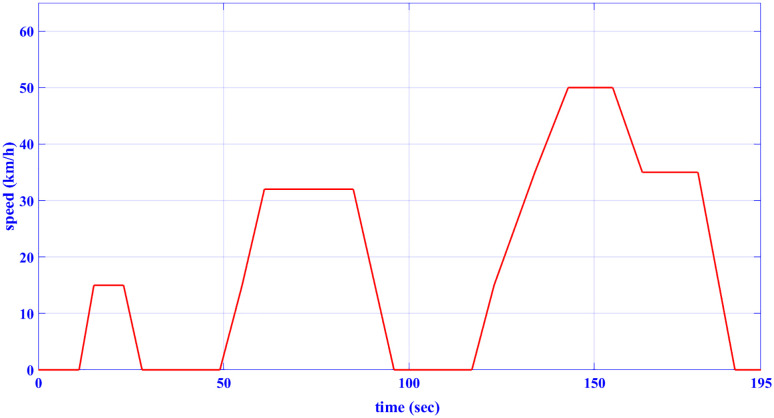
ECE-15 drive cycle [31].

**Fig 3:**
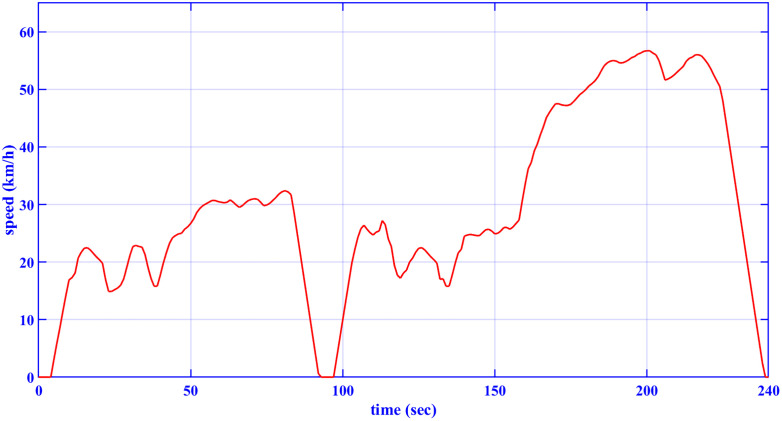
IM240 drive cycle [42].

The drive cycles are modeled in terms of their speed-time graph. The adopted drive cycles in this paper are the European drive cycle (ECE-15), Fig 2 [31], and the customized inspection and maintenance drive cycle (IM240), Fig 3 [42]. Table 1 indicates the characteristics of the two drive cycles.

**Table 1:** Characteristics of the drive cycles.

Features	ECE-15	Custom IM240
Distance	0.9941 km	3.1 km
Total time	195 s	240 s
Average speed (incl. stops)	18.35 km/h	29.4 km/h
Maximum speed	50 km/h	57.6 km/h

#### 2.2 EV model.

**Fig 4:**
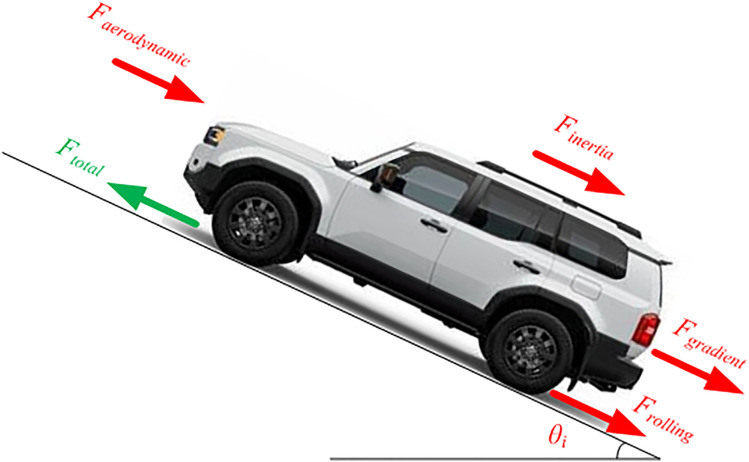
Forces affecting the total force of an electric vehicle.

The input of the vehicle model is the velocity obtained from the drive cycle. The model outputs are the required vehicle torque and the required motor speed. The vehicle torque can be obtained by calculating the total force. The total force is the resultant of the forces affecting the vehicle. These forces are aerodynamic force, rolling force, gradient force, and inertia force, as shown in Fig 4.

The aerodynamic force, *Fairodynamic*, can be expressed in the following formula [2,34,39]:


$$F_{airodynamic}=\frac{\text{1}}{\text{2}}\rho_{a}C_{ad}A_{fa}(V_{v}+V_{wind}{)}^{2}$$ (1)

Where *ρa* is the air density in kg/m3, *Cad* is the aerodynamic drag coefficient, *Afa* is the vehicle frontal area in m2, *Vv* is the vehicle velocity in m/s, and *Vwind* is the wind speed in m/s. The tyre rolling force, *Frolling*, takes the following form [2,34,39]:


$$F_{rolling}=m_{v}g_{a}C_{rr}\cos\theta_{i}$$ (2)

Where *mv* is the vehicle mass in kg, ga is the gravitational acceleration, *Crr* is the tyre rolling resistance coefficient, and *θi* is the vehicle inclination angle in degrees. The following formula indicates the gradient force, *Fgradient* [2,34,39]:


$$F_{gradient}=m_{v}g_{a}\sin\theta_{i}$$ (3)

The inertia force, *Finertia*, can be expressed in the following formula [2,34,39]:


$$F_{inertia}=m_{v}a_{v}$$ (4)

Where *av* is the vehicle acceleration. Thus, the total vehicle force can be obtained from the following formula [2,34,39]:


$$F_{total}=F_{aerodynamic}+F_{rolling}+F_{gradient}+F_{inertia}$$ (5)

Consequently, the required motor torque can be obtained from [2,34,39]:


$$T_{req}=F_{total}R_{wheel}/G_{R}$$ (6)

Where *Rwhee*l is the vehicle wheel radius, and *GR* is the gear ratio. Also, the motor required speed can be obtained from [2,34,39]:


$$\omega_{req}=G_{R}V_{v}/R_{wheel}$$ (7)

The EV required power can thus be obtained from:


$$P_{req}=T_{req}\omega_{req}$$ (8)

In addition to this, the required energy can be expressed as:


$$E_{req}=\int P_{req}dt$$ (9)

#### 2.3 5-Phase VSI model.

The VSI is represented through the voltages (*va* to *ve*), which are influenced by switching functions of the VSI [10,41].


$$\left[\begin{array}{c} {}*20cv_{a}\\ v_{b}\\ v_{c}\\ v_{d}\\ v_{e} \end{array}\right]=\frac{V_{dc}}{\text{5}}\left[\begin{array}{ccccc} {}*20c4 & -1 & -1 & -1 & -1\\ -1 & 4 & -1 & -1 & -1\\ -1 & -1 & 4 & -1 & -1\\ -1 & -1 & -1 & 4 & -1\\ -1 & -1 & -1 & -1 & 4 \end{array}\right].\left[\begin{array}{c} {}*20cs_{a}\\ s_{b}\\ s_{c}\\ s_{d}\\ s_{e} \end{array}\right]$$ (10)

In Eq. (10), Vdc denotes the battery voltage. The switching functions, labeled as Sa to Se, indicate the different VSI switching states. A switching function takes the value of one when the upper switch in a leg is ON, and zero when the lower switch of this leg is OFF. There are 32 switching states. The MPC provides the optimal switching state that gives a minimized cost function. According to the selected state from the 32 states, gate pulses are provided to ten switches of the VSI.

#### 2.4 5-phase IPMSM model.

The 5-phase IPMSM is characterized by the aiding of the direct-quadrature analysis in a synchronously rotating reference frame. The transformations from voltage ABCDE to DQ are detailed in [10,41,43].


$$\left[\begin{array}{c} {}*20cv_{d1}\\ v_{q1}\\ v_{d3}\\ v_{q3}\\ v_{0} \end{array}\right]=\frac{\text{2}}{\text{5}}\left[\begin{array}{ccccc} {}*20c\cos\theta & \cos(\theta -\alpha ) & \cos(\theta -2\alpha ) & \cos(\theta +2\alpha ) & \cos(\theta +\alpha )\\ -\sin\theta & -\sin(\theta -\alpha ) & -\sin(\theta -\alpha ) & -\sin(\theta +2\alpha ) & -\sin(\theta +\alpha )\\ \cos3\theta & \cos3(\theta -\alpha ) & \cos3(\theta -2\alpha ) & \cos3(\theta +2\alpha ) & \cos3(\theta +\alpha )\\ -\sin3\theta & -\sin3(\theta -\alpha ) & -\sin3(\theta -2\alpha ) & -\sin3(\theta +2\alpha ) & -\sin3(\theta +\alpha )\\ \frac{\text{1}}{\sqrt{2}} & \frac{\text{1}}{\sqrt{2}} & \frac{\text{1}}{\sqrt{2}} & \frac{\text{1}}{\sqrt{2}} & \frac{\text{1}}{\sqrt{2}} \end{array}\right].\left[\begin{array}{c} {}*20cv_{a}\\ v_{b}\\ v_{c}\\ v_{d}\\ v_{e} \end{array}\right]$$ (11)

Where the value of α is 2π/5, *vd1* and *vq1* are the DQ fundamental stator voltages, *vd3* and *vq3* are the 3rd harmonic DQ stator voltages, and θ represents the rotor position angle.

To transform from the DQ to the ABCDE quantities, the following equation can be used [10,41]:


$$\left[\begin{array}{c} {}*20ci_{as}\\ i_{bs}\\ i_{cs}\\ i_{ds}\\ i_{es} \end{array}\right]=\left[\begin{array}{ccccc} {}*20c\cos\theta & -\sin\theta & \cos3\theta & -\sin3\theta & \frac{\text{1}}{\sqrt{2}}\\ \cos(\theta -\alpha ) & -\sin(\theta -\alpha ) & \cos3(\theta -\alpha ) & -\sin3(\theta -\alpha ) & \frac{\text{1}}{\sqrt{2}}\\ \cos(\theta -2\alpha ) & -\sin(\theta -2\alpha ) & \cos3(\theta -2\alpha ) & -\sin3(\theta -2\alpha ) & \frac{\text{1}}{\sqrt{2}}\\ \cos(\theta +2\alpha ) & -\sin(\theta +2\alpha ) & \cos3(\theta +2\alpha ) & -\sin3(\theta +2\alpha ) & \frac{\text{1}}{\sqrt{2}}\\ \cos(\theta +\alpha ) & -\sin(\theta +\alpha ) & \cos3(\theta +\alpha ) & -\sin3(\theta +\alpha ) & \frac{\text{1}}{\sqrt{2}} \end{array}\right].\left[\begin{array}{c} {}*20ci_{d1}\\ i_{q1}\\ i_{d3}\\ i_{q3}\\ i_{0} \end{array}\right]$$ (12)

Where *id1* and *iq1* are the DQ fundamental stator currents, and *id3* and *iq3* are the DQ 3rd harmonic stator currents.

The differential equation of the 5-phase IPMSM can be expressed as follows [41]:


D[I]=[LL][V]−[LR].[I]−ω[Lλ].[I]+ω[LG].[I] (13)

Where D is the differentiator d/dt,$[I]=[\begin{array}{cccc} {}*20ci_{d1} & i_{q1} & i_{d3} & i_{q3} \end{array}{]}^{T}$,$[V]=[\begin{array}{cccc} {}*20cv_{d1} & v_{q1} & v_{d3} & v_{q3} \end{array}{]}^{T}$,


$$[LL]=\left[\begin{array}{cccc} {}*20c\frac{-L_{d3}}{L_{m13}^{2}-L_{d1}L_{d3}} & 0 & \frac{L_{m13}}{L_{m13}^{2}-L_{d1}L_{d3}} & 0\\ 0 & \frac{-L_{q3}}{L_{m13}^{2}-L_{q1}L_{q3}} & 0 & \frac{L_{m13}}{L_{m13}^{2}-L_{q1}L_{q3}}\\ \frac{L_{m13}}{L_{m13}^{2}-L_{d1}L_{d3}} & 0 & \frac{-L_{d1}}{L_{m13}^{2}-L_{d1}L_{d3}} & 0\\ 0 & \frac{L_{m13}}{L_{m13}^{2}-L_{q1}L_{q3}} & 0 & \frac{-L_{q1}}{L_{m13}^{2}-L_{q1}L_{q3}} \end{array}\right]$$

$$[LR]=\left[\begin{array}{cccc} {}*20c\frac{-L_{d3}R_{s}}{L_{m13}^{2}-L_{d1}L_{d3}} & 0 & \frac{L_{m13}R_{s}}{L_{m13}^{2}-L_{d1}L_{d3}} & 0\\ 0 & \frac{-L_{q3}R_{s}}{L_{m13}^{2}-L_{q1}L_{q3}} & 0 & \frac{L_{m13}R_{s}}{L_{m13}^{2}-L_{q1}L_{q3}}\\ \frac{L_{m13}R_{s}}{L_{m13}^{2}-L_{d1}L_{d3}} & 0 & \frac{-L_{d1}R_{s}}{L_{m13}^{2}-L_{d1}L_{d3}} & 0\\ 0 & \frac{L_{m13}R_{s}}{L_{m13}^{2}-L_{q1}L_{q3}} & 0 & \frac{-L_{q1}R_{s}}{L_{m13}^{2}-L_{q1}L_{q3}} \end{array}\right]$$

$$[LG]=\left[\begin{array}{cccc} {}*20c0 & \frac{3L_{m13}^{2}-L_{d3}L_{q1}}{L_{m13}^{2}-L_{d1}L_{d3}} & 0 & \frac{3L_{m13}L_{q3}-L_{d3}L_{m13}}{L_{m13}^{2}-L_{d1}L_{d3}}\\ \frac{L_{d1}L_{q3}-3L_{m13}^{2}}{L_{m13}^{2}-L_{q1}L_{q3}} & 0 & \frac{L_{m13}L_{q3}-3L_{d3}L_{m13}}{L_{m13}^{2}-L_{q1}L_{q3}} & 0\\ 0 & \frac{L_{m13}L_{q1}-3L_{d1}L_{m13}}{L_{m13}^{2}-L_{d1}L_{d3}} & 0 & \frac{L_{m13}^{2}-3L_{d1}L_{q3}}{L_{m13}^{2}-L_{d1}L_{d3}}\\ \frac{3L_{m13}L_{q1}-L_{d1}L_{m13}}{L_{m13}^{2}-L_{q1}L_{q3}} & 0 & \frac{3L_{d3}L_{q1}-L_{m13}^{2}}{L_{m13}^{2}-L_{q1}L_{q3}} & 0 \end{array}\right]$$

$$[L\lambda ]=\left[\begin{array}{c} {}*20c0\\ \frac{3\lambda_{3m}L_{m13}-\lambda_{1m}L_{q3}}{L_{m13}^{2}-L_{q1}L_{q3}}\\ 0\\ \frac{\lambda_{1m}L_{m13}-3\lambda_{3m}L_{q1}}{L_{m13}^{2}-L_{q1}L_{q3}} \end{array}\right]$$

Where *Rs* represents the per-phase resistance, ω denotes electrical angular speed, *Ld1* and *Lq1* represent the DQ fundamental self-inductances, *Ld3* and *Lq3* represent the DQ 3rd harmonic self-inductances, *Lm13* is the mutual inductance, and *λ1m*, *λ3m* are the PM fluxes for the fundamental and 3rd component.

The motor torque can be expressed as [10,41,43]:


$$T_{e}=\frac{\text{5}}{\text{2}}\frac{p}{\text{2}}[(L_{d1}-L_{q1})i_{d1}i_{q1}+2L_{m13}(i_{d1}i_{q3}-i_{q1}i_{d3})+3(L_{d3}-L_{q3})i_{d3}i_{q3}+(\lambda_{1m}i_{q1}+3\lambda_{3m}i_{q3})]$$ (14)

Where p is the number of poles.

The mechanical equation can be expressed as [41]:


$$D\omega_{m}=\frac{T_{e}-T_{l}(\omega_{m})}{J}$$ (15)

where J is the inertia, and $T_{l}(\omega_{m})$ is given by:


$$T_{l}(\omega_{m})=T_{L}+T_{fw}$$ (16)

Where $T_{L}$ is the load torque and $T_{fw}$ is the friction and windage torque.

#### 2.5 5-phase IPMSM MTPA operating model.

To achieve maximum efficiency, the 5-phase IPMSM operates at MTPA. When Lm13 is neglected, the fundamental and third-harmonic DQ currents, that give MTPA, can be obtained from [41]:


$$i_{d1}=\frac{\lambda_{1m}}{2(L_{q1}-L_{d1})}-\sqrt{\frac{\lambda_{1m}^{2}}{4{\left(L_{q1}-L_{d1}\right)}^{2}}+i_{q1}^{2}}$$ (17)

$$i_{q1}=\frac{T_{e1}}{\frac{\text{5}}{\text{2}}\frac{p}{\text{2}}[(L_{d1}-L_{q1})i_{d1}+\lambda_{1m}]}$$ (18)

Also, the 3rd harmonic direct and quadrature currents can be obtained from [41,44]:


$$i_{d3}=k\sqrt{i_{d1}^{2}+i_{q1}^{2}}\sin\{3[\tan^{-1}(\frac{i_{d1}}{i_{q1}})\}$$ (19)

$$i_{q3}=k\sqrt{i_{d1}^{2}+i_{q1}^{2}}\cos\{3[\tan^{-1}(\frac{i_{d1}}{i_{q1}})\}$$ (20)

## 3 Model predictive control technique

MPC is composed of a plant model and an optimizer. The main objective of MPC is the selection of the best arrangement of inputs for the system by forecasting its behavior in the future. The forecasts are generated by the plant model, which relies on past states to predict the next ones. At every sampling period, the optimizer utilizes the forecasted states and the required trajectory to solve the problem of optimization over the forecasting horizon, thus determining the optimized group of inputs for future executions.

To effectively achieve MPC, it is essential to discretize the 5-phase IPMSM model. Therefore, eqn. (13) is transformed into its discrete form as follows [41]:


$$[I(k+1)]=[I(k)]+T_{s}\{[LL][V]-[LR].[I(k)]-\omega [L\lambda ].[I(k)]+\omega [LG].[I(k)\}$$ (21)

Where Ts is the sampling period.

The main objective of the MPC is to minimize torque error. The torque error can be minimized by minimizing the DQ currents errors. Consequently, the cost function (CF) that minimizes the DQ currents errors can be written in the following form [41]:


$$C.F=[i_{d1r}-i_{d1}(k+1){]}^{2}+[i_{q1r}-i_{q1}(k+1){]}^{2}+[i_{d3r}-i_{d3}(k+1){]}^{2}+[i_{q3r}-i_{q3}(k+1){]}^{2}$$ (22)

Here, (*id1r*, *iq1r*, *id3r*, and *iq3r*) denote the reference DQ currents. They can be calculated with the aid of equations (17), (18), (19), and (20). The optimum 5-phase VSI switching functions are selected according to the lowest CF.

## 4 Proposed adaptive PI-ANN controller

The mathematical formulation of a conventional PI controller is expressed as follows:


$$u\left(t\right)=\;K_{P}e_{t}\left(t\right)+\;K_{i}\int e_{t}\left(t\right)\;dt$$ (23)

Here, $K_{P}$ is the proportional gain and $\;K_{i}$ is the integral gain. $e_{t}\left(t\right)$ represents the speed error signal, and u(t) is the controller output. These PI parameters are typically set to constant values using methods such as Ziegler-Nichols’ first tuning method [45].

**Fig 5:**
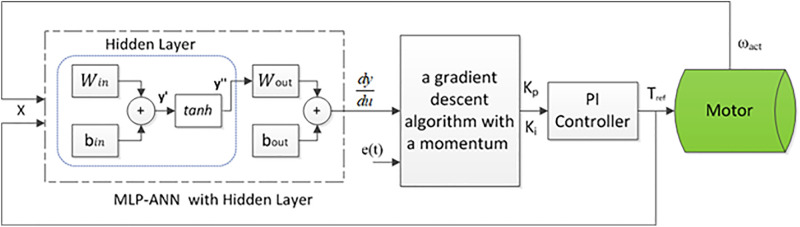
The proposed adaptive PI-ANN controller.


ANN provides a data-driven approach to model system behavior through learning mechanisms based on error minimization criteria. In this study, a multi-layer perceptron (MLP) ANN was implemented in an offline mode for training. The MLP architecture consists of a single hidden layer with 20 neurons, as shown in Fig 5. The model of the MLP-ANN and its associated learning error criteria are represented as follows [45]:

$$\hat{y}=W_{out}^{T}\tanh\left(W_{in}^{T}X+b_{in}\right)+b_{out}$$ (24)

$$E=\frac{\text{1}}{\text{2}}\;\sum {\left(e\right)}^{2}=\frac{\text{1}}{\text{2}}\;\sum {\left(y-\hat{y}\right)}^{2}$$ (25)

Where X is the input vector, y is the actual output, $\hat{y}$ is the predicted output, e is the error signal, and E denotes MSE used as the CF. The terms $W_{in}$, $W_{out}$, $b_{in}$, and $b_{out}$ represent the weights and biases of the ANN.

The proposed adaptive PI-ANN control strategy leverages the MLP-ANN model [45]. By utilizing the Jacobian matrix of the system, the $K_{P}$ and $\;K_{i}$ are updated in each iteration of the control loop. The block diagram of the suggested control algorithm is depicted in Fig 5. This approach incorporates the Jacobian matrix to compute the gradient vector, enabling the adjustment of the PI parameters using the chain rule of the backpropagation algorithm, as shown below:


$$\frac{dy}{du}≅\frac{d\hat{y}}{du}=W_{in}^{T}W_{out}^{T}\frac{dy^{\prime \prime }}{dy^{\prime }}$$ (26)

$$\frac{dE}{dK_{P,i}}=\frac{dE}{de}\frac{de}{d\hat{y}}\frac{d\hat{y}}{dX}\frac{dX}{du}\frac{du}{dK_{P,i}}$$ (27)

Here $\frac{dy}{du}≅\frac{d\hat{y}}{du}$ represents the Jacobian matrix of the ANN, $\frac{dy^{\prime \prime }}{dy^{\prime }}=1-tanch^{2}\left(y^{\prime }\right)$ is the differentiation of the function of activation, $\frac{dE}{dK_{P,i}}$ are the derivatives of the CF w.r.t the PI parameters, $\frac{dE}{de}$ is the derivative of the CF w.r.t the error, $\frac{de}{d\hat{y}}$ is determined as −1, $\frac{du}{dK_{P,i}}$ are the derivatives of the control signal u(t) with respect to the PI parameters, $\frac{du}{dK_{P}}=e_{t}\left(t\right)$ and $\frac{du}{dK_{i}}=\int {\mathrm{\backslash nolimits}}_{0}^{\infty }e_{t}\left(t\right)\;dt$, and $e_{t}\left(t\right)$ is the closed-loop error signal.

To adaptively update the PI parameters, a gradient descent algorithm with a momentum term [45] is employed. This iterative update rule is given as follows:


$$K_{P,i}\left(n\right)=K_{P,i}\left(n-1\right)-\alpha VK_{P,i}\left(n\right)$$ (28)

$$VK_{P,i}\left(n\right)=\beta \nabla_{K_{P,i}}\left(n-1\right)+\left(1-\beta \right)\nabla_{K_{P,i}}\left(n\right)$$ (29)

**Fig 6:**
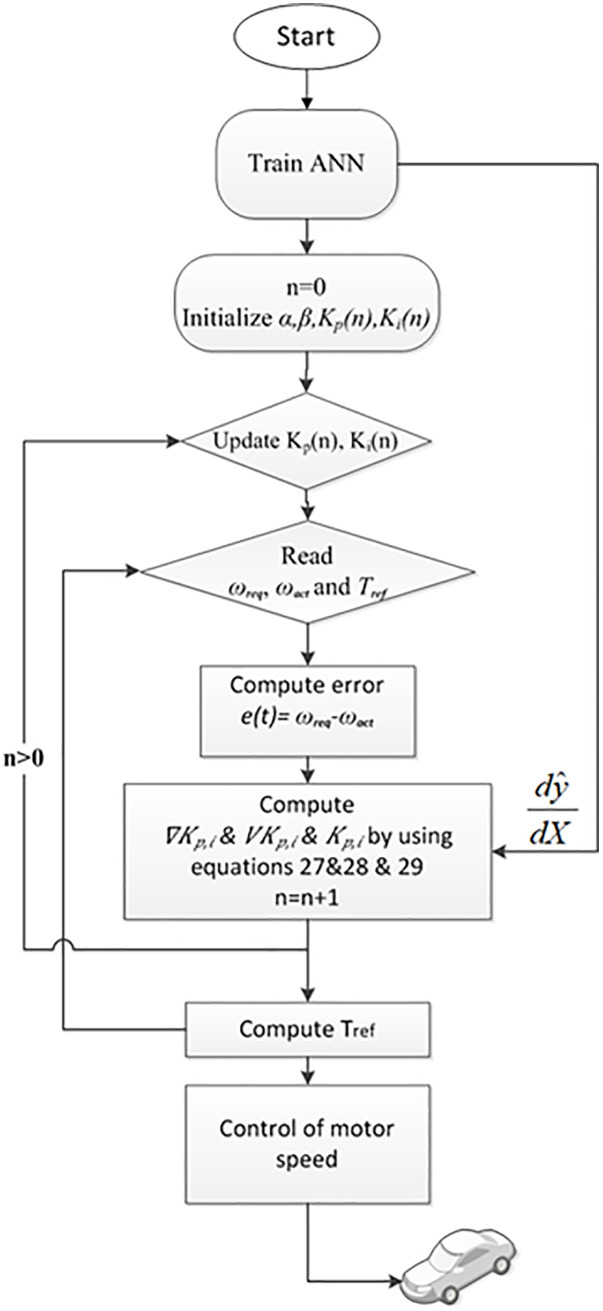
Flowchart Proposed Adaptive PI Control Algorithm.

In this equation, $K_{P,i}$ are the PI parameters, $\nabla_{K_{P,i}}=\frac{du}{dK_{P,i}}$ is the gradient, n is the iteration number, α is the learning rate, and β is the momentum coefficient. The flowchart for the suggested algorithm is shown in Fig 6.

## 5 Transit search optimization technique

A TSO algorithm [46] model is achieved for optimal tuning of the PI controller.

### (5.1) Objective

The optimization objective concentrates on minimizing the speed error by tuning its controllers’ gains. The controller contains two gains (proportional gain KP and the integral gain KI). The gains have a lower limit [0 0] and upper limit [100 100].


Target=min(error) (30)

$$error\;=\int {\left(\omega_{actual\;}-\omega_{ref}\right)}^{2}dt$$

### (5.2) Transit search algorithm

**Fig 7:**
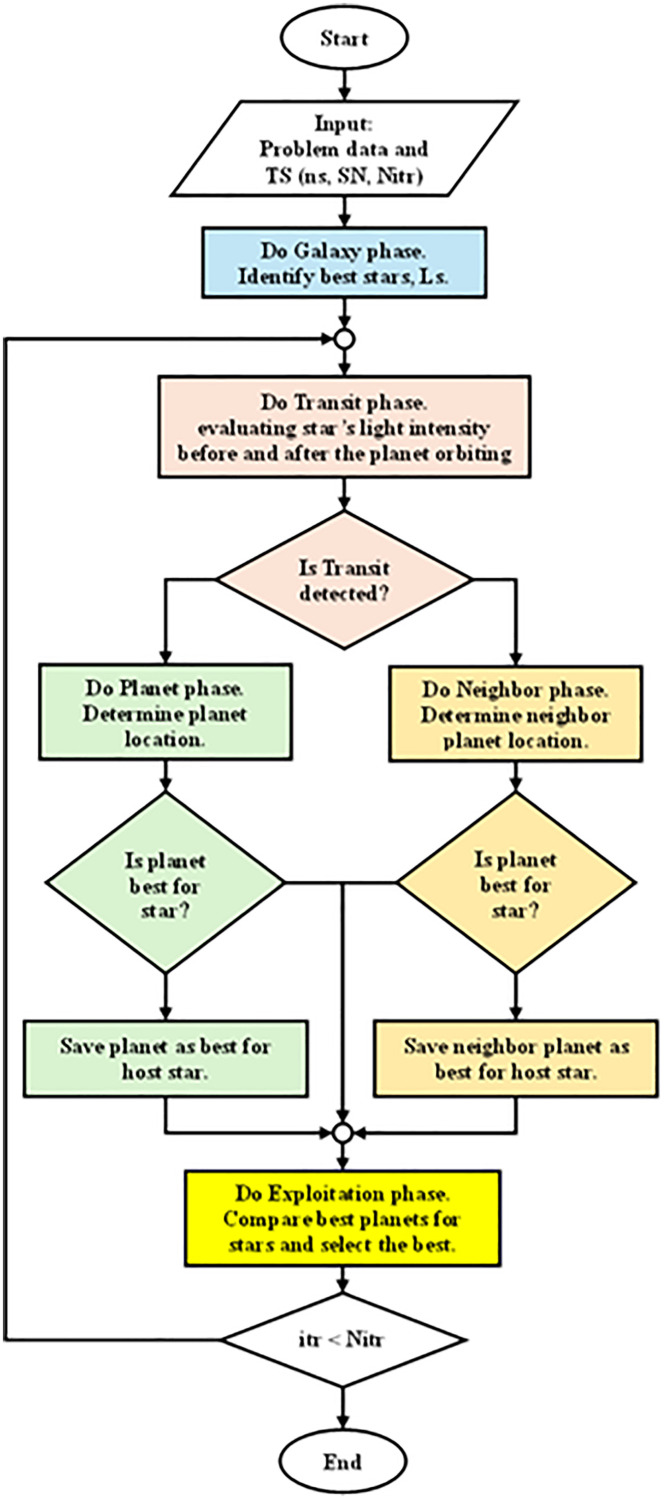
Flowchart of the TSO algorithm.

The flowchart of the TSO algorithm steps, which is used in this work, is shown in Fig 7. The optimization process is composed of five split phases in the TSO algorithm. First, Galaxy phase: executes for initialization by assigning a galaxy center in a random way to determine a galaxy and its habitable zones and determine the proper position of the star. Second, the Transit phase starts by assessing the change in the star’s intensity of light during the rotation of the planet. Third, Planet phase: when a planet transit is detected, the initial position of the specific planet is calculated. In every iteration, the planet identified with better fitness is marked. Fourth, Neighbor phase: executes when transit isn’t detected by analyzing the neighboring planet and replacing the present planet in case it has better fitness. Finally, the phase of exploitation determines the best planet for every star by studying many scenarios of the imported existing knowledge from the previous phases.

## 6 Results

Several simulation results are obtained to ensure the validity of the presented control methodology for the EV drive system under consideration using the two test driving cycles, ECE-15 and custom IM240. The vehicle parameters are given in Table 2 [34]. The parameters of the motor are given in Table 3 [10]. The inclination angle, θi, is taken to be 25°. The offline tuning gains, kp and ki for the PI controller, are obtained at motor rated condition using Transit Search optimization (TSO) technique [46] and found to be 10.3486 and 25.0843, respectively.

**Table 2:** Electric vehicle parameters.

Parameter	Value
Kerb and passenger weight (mv), kg	158
Frontal area (Afa), m2	0.875
Wheel radius (Rwheel), m	0.23
Aerodynamic drag coefficient (Cad)	0.22
Gear ratio (GR)	3.1:1
*Tyre rolling resistance coefficient (Crr)*	0.03

**Table 3:** 5-Table 4Table 5ph IPMSM parameters.

Parameter	Value
poles (p)	4 poles
Nominal power	12 kW
Nominal Speed	1800 rpm
Base speed	5400 rpm
Nominal Torque	63.662 Nm
*Rs*	0.389 Ω
*Ld1*	2.7 mH
*Lq1*	9.6 mH
*Ld3*	1.1 mH
*Lq3*	2 mH
*Lm13*	0 mH
*λ1m*	0.11 WbT
*λ3m*	0.0012 WbT
Motor moment of inertia	0.0036 kg.m2
Total moment of inertia	0.14 kg.m2
Connection	Star

**Fig 8:**
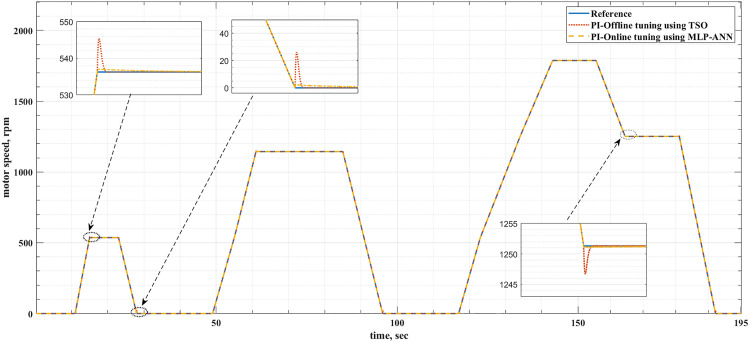
The reference and actual motor speeds for the ECE-15 drive cycle when offline and online PI tuning are used.

**Fig 9:**
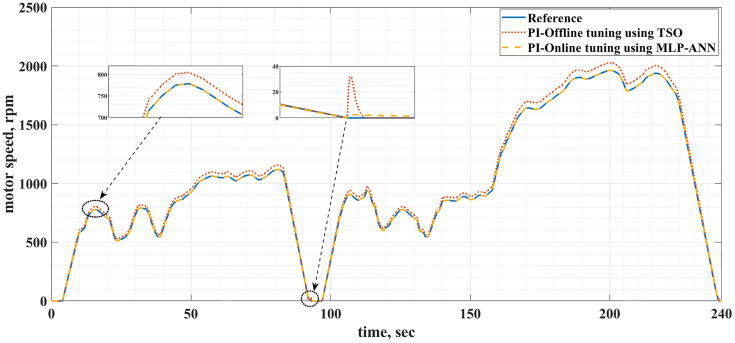
The reference and actual motor speeds for the custom IM240 drive cycle when offline and online PI tuning are used.

**Fig 10:**
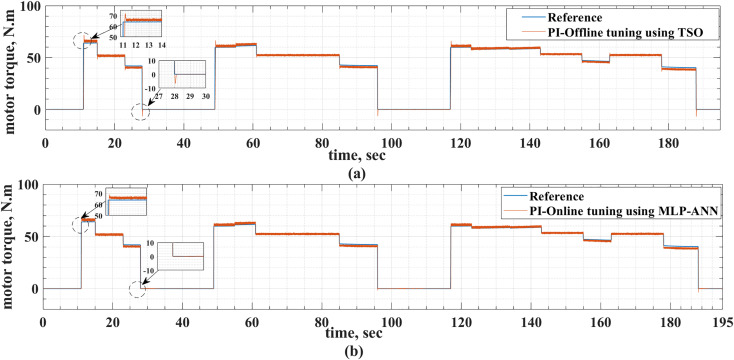
The reference and actual motor torque for the ECE-15 drive cycle when the offline and online PI tuning methods are used.

**Fig 11:**
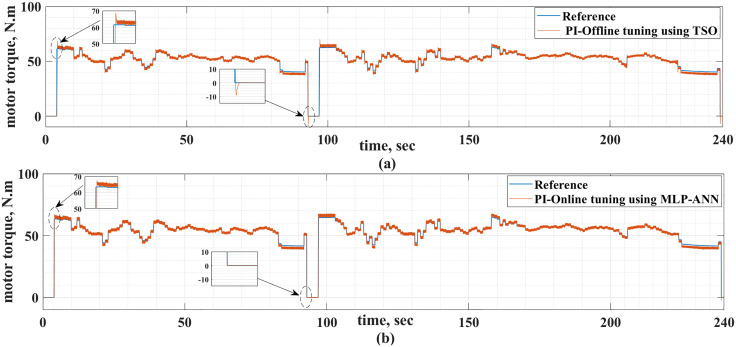
The reference and actual motor torque for the custom IM240 drive cycle when the offline and online PI tuning methods are used.

Figs 8 and 9 show the reference and actual motor speeds for both drive cycles when the PI controller is tuned either offline using TSO or online using MLP-ANN. It can be noticed that there are many overshoots in the motor speed when the offline tuning is used. Therefore, the online tuning has better speed response regardless of the drive cycle type. The speed overshoots are due to the overshoots and undershoots in the motor torque, as shown in Figs 10 and 11. Tables 4 and 5 summarize the difference between the offline and online tuning for the PI controller in the EV drive system under consideration for both drive cycles. It can be concluded from these tables that the utilization of the MLP-ANN online in the drive system, tested by the two drive cycles, provides lower values of MSE, IAE, and percentage overshoot in the motor speed compared with the offline tuning using TSO, i.e., better response.

**Table 4:** Speed response comparison between the PI-offline tuning using TSO and PI-online tuning using MLP-ANN when the ECE-15 drive cycle is used.

PI Tuning Method	MSE	IAE	Percentage Overshoot (%)
Offline tuning using TSO	0.009809	0.012091	1.658%
MLP-ANN online tuning	0.001737	0.002970	0.1366%

**Table 5:** Speed response comparison between the PI-offline tuning using TSO and PI-online tuning using MLP-ANN when the custom IM240 drive cycle is used.

PI Tuning Method	MSE	IAE	Percentage Overshoot (%)
PI-offline tuning using TSO	0.007134	0.010105	3.334190%
PI-online tuning using MLP-ANN	0.001258	0.002529	0.006423%

**Fig 12:**
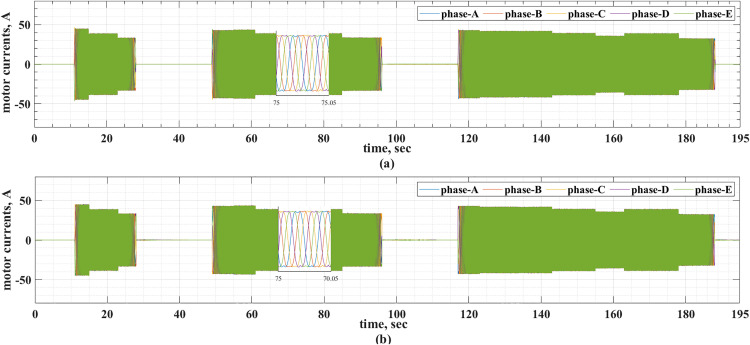
The motor currents for the ECE-15 drive cycles when (a) offline and (b) online PI tuning are used.

**Fig 13:**
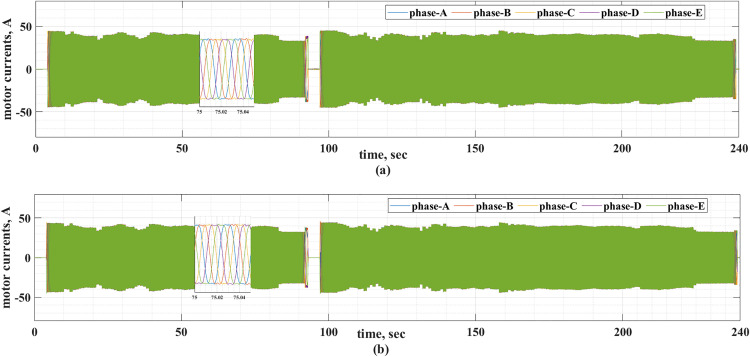
The motor currents for the custom IM240 drive cycle when (a) offline and (b) online PI tuning are used.

**Fig 14:**
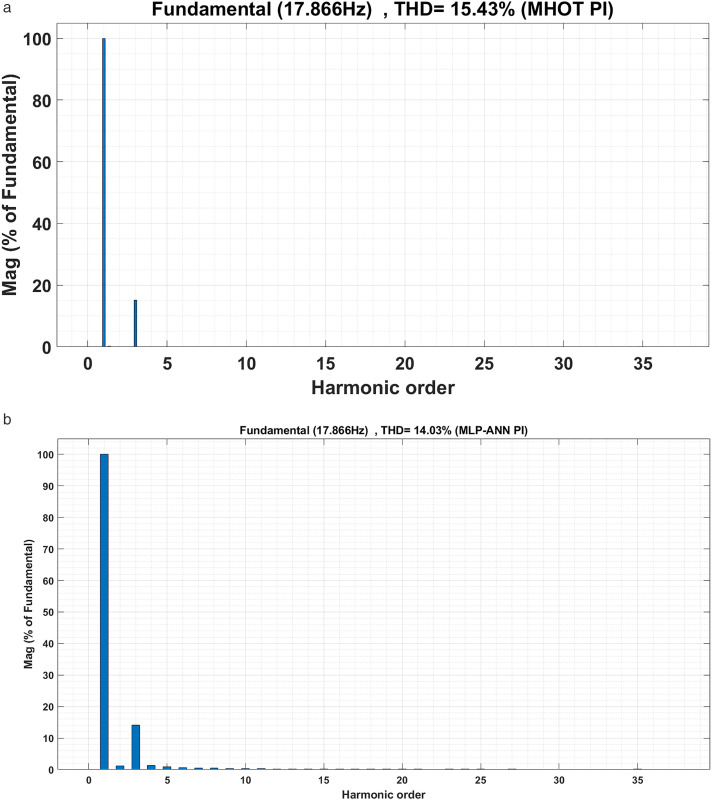
The motor currents THDs for the ECE-15 drive cycles when (a) offline and (b) online PI tuning are used.

**Fig 15:**
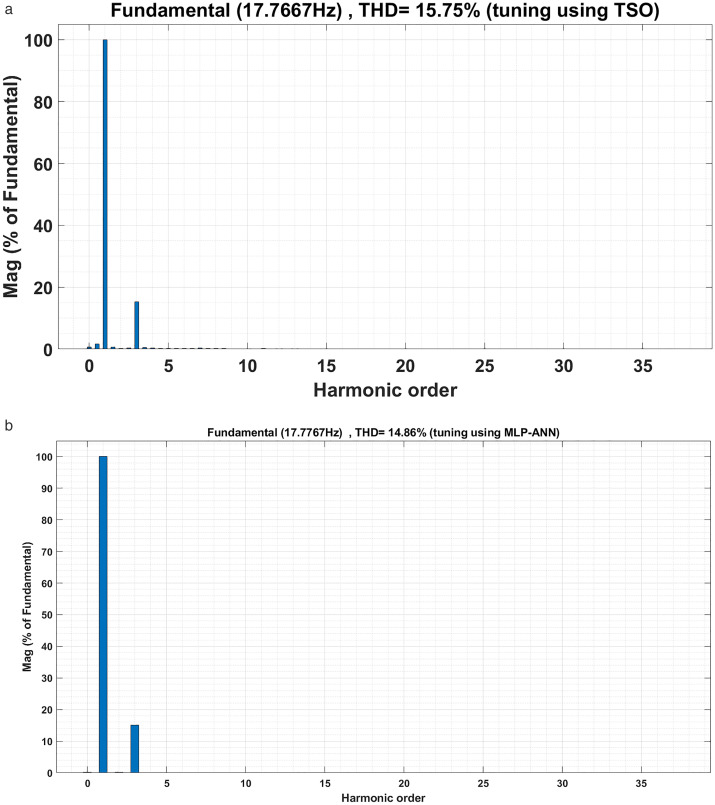
The motor currents THDs for the custom IM240 drive cycles when (a) offline and (b) online PI tuning are used.

The motor currents for the two drive cycles under consideration are shown in Figs 12 and 13. It can be noticed that the motor currents are almost sinusoidal. The total harmonic distortions (THDs) of the motor currents are shown in Figs 14 and 15 for the two drive cycles. It can be noticed from these figures that the THDs are slightly lower when the online running is used compared with the offline tuning.

**Fig 16:**
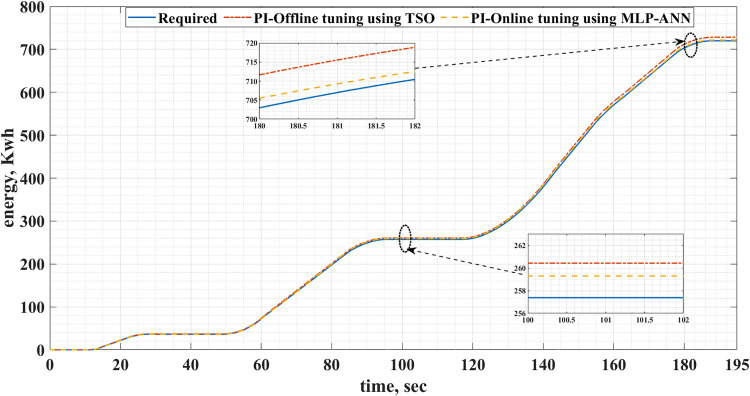
The required and actual EV energy consumed for the ECE-15 drive cycles when offline and online PI tuning are used.

**Fig 17:**
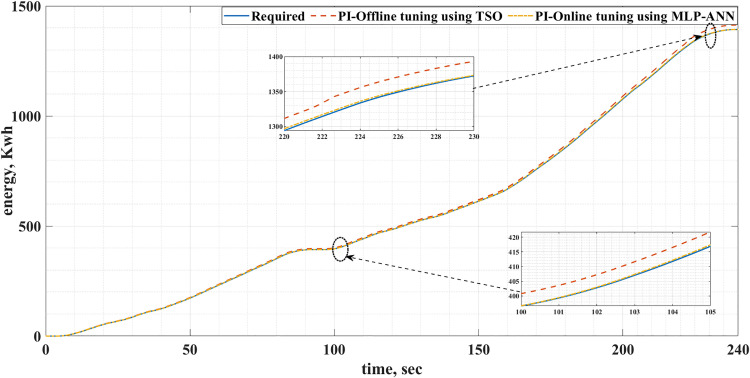
The required and actual EV energy consumed for the custom IM240 drive cycles when offline and online PI tuning are used.

Figs 16 and 17 show the required EV consumed energy and the actual energy consumed when offline and online tuning are used for the two driving cycles. It is clear from these figures that the energy consumed for online tuning using MLP-ANN is less than that of offline tuning using TSO. This is also shown in detail in Table 6. Consequently, the use of the MLP-ANN online tuning for the PI controller in the EVDS under consideration results in energy saving. It should be noticed that these energy savings happened during small time intervals, 195s for the ECE-15 drive cycle and 240s for the IM240 drive cycle. Consequently, the quantity of energy saved will be greater for a longer time. This energy saving results in cost reduction of charging the batteries and increasing their lifetime.

**Table 6:** Energy saving comparison between the offline and ANN online tuning for the two drive cycles.

Drive cycle	EV required Energy, kWh	Energy Consumption in case of Offline tuning using Transit Search, kWh	Energy Consumption in case of ANN online tuning, kWh	Energy Saving, kWh
ECE-15	719.984	732.458	728.074	4.39
Custom IM240	1392.592	1412.721	1392.582	20.13

**Fig 18:**
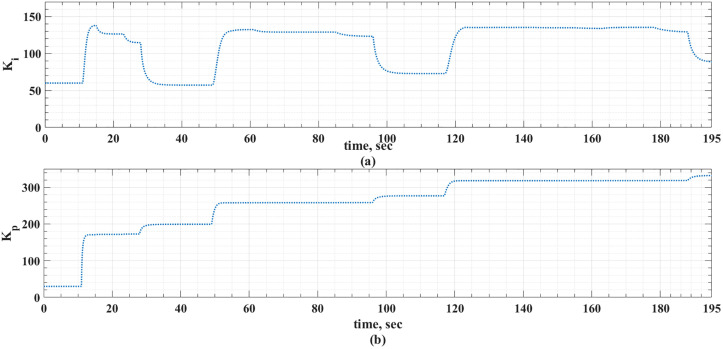
The PI-online tuning gains using MLP-ANN for the ECE-15 drive cycles.

**Fig 19:**
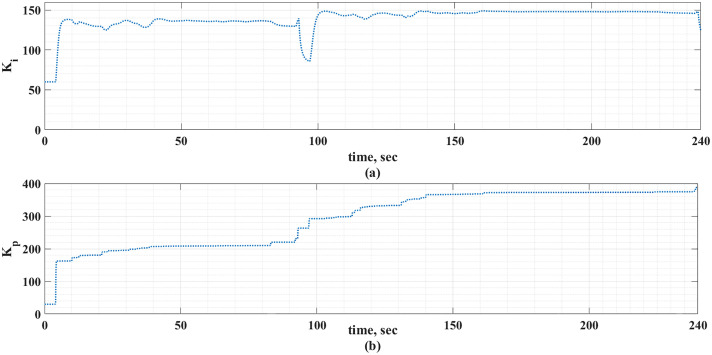
The PI-online tuning gains using MLP-ANN for the custom IM240 drive cycle.

The MLP-ANN online tuning gains of the PI controller for the two driving cycles under consideration are shown in Figs 18 and 19.

## 7 Conclusion

In this study, an efficient EV speed tracking control methodology is presented. This methodology is based on using an MLP-ANN online tuning for a PI controller. The adaptation of the five-phase IPMSM and MPC enhanced the efficiency and the reliability of the EV drive system. Two drive cycles, ECE-15 and custom IM240, are used to test the proposed control methodology of the EV drive system. A set of compared results between the offline tuning, using a recent metaheuristic optimization technique (TSO), and MLP-ANN online tuning for the PI controller used in the system under consideration. The result of the comparison showed that online tuning using MLP-ANN gives superior performance. The online tuning gives lower values of MSE, IAE, and percentage overshoot in the motor speed compared with the offline tuning method for the two test drive cycles adopted in this research. For the ECE-15 drive cycle, the MSE, IAE, and overshoot percentage are 0.001737, 0.002970, and 0.1366%, respectively, while for the IM240 drive cycle, the values are 0.001258, 0.002529, and 0.006423%. Additionally, online tuning using MLP-ANN attains energy saving, with a maximum energy saving of 20.13 kWh compared to the conventional approach, which will be large over a long time, which in turn results in cost reduction of charging the batteries and increasing their lifetime. Finally, the THDs of the motor currents are slightly lower when the online tuning using MLP-ANN is used compared with the offline tuning using TSO. However, compared with offline tuning, online tuning has higher computational costs and potential delays in real-time applications. In addition to this, the implementation of online tuning algorithms is more complex.
